# Healthcare resource use and economic burden attributable to respiratory syncytial virus in the United States: a claims database analysis

**DOI:** 10.1186/s12913-018-3066-1

**Published:** 2018-04-20

**Authors:** Caroline Amand, Sabine Tong, Alexia Kieffer, Moe H. Kyaw

**Affiliations:** 1 Sanofi, Chilly-Mazarin, France; 2 IVIDATA Stats, Levallois-Perret, France; 3 Sanofi Pasteur, Lyon, France; 4 Sanofi Pasteur, Swiftwater, PA 18370 USA

**Keywords:** RSV, Healthcare resource use, Healthcare costs, Economic burden

## Abstract

**Background:**

Despite several studies that have estimated the economic impact of Respiratory Syncytial Virus (RSV) in infants, limited data are available on healthcare resource use and costs attributable to RSV across age groups. The aim of this study was to quantify age-specific RSV-related healthcare resource use and costs on the US healthcare system.

**Methods:**

This retrospective case-control study identified patients aged ≥1 year with an RSV event in the Truven Health Marketscan® Commercial Claims and Encounters and Medicare Supplemental and Coordination of Benefits databases between August 31, 2012 and August 1, 2013. RSV patients were matched 1:1 with non-RSV controls for age, gender, region, healthcare plan and index date (*n* = 11,432 in each group). Stratified analyses for healthcare resource use and costs were conducted by age groups. RSV-attributable resource use and costs were assessed based on the incremental differences between RSV cases and controls using multivariate analysis.

**Results:**

RSV patients had a higher healthcare resource use (hospital stays, emergency room/urgent care visits, ambulatory visits and outpatient visits) than non-RSV matched controls for all age groups (all *p* < 0.0001), particularly in the elderly age groups with RSV (1.9 to 3 days length of stay, 0.4 to 0.5 more ER/UC visits, 0.7 to 2.7 more ambulatory visits, 12.1 to 18.6 more outpatient visits and 9.5 to 14.6 more prescriptions than elderly in the control groups). The incremental difference in adjusted mean annual costs between RSV and non-RSV controls was higher in elderly (≥65; $12,030 to $23,194) than in those aged < 65 years ($2251 to $5391). Among children, adjusted costs attributable to RSV were higher in children aged 5–17 years ($3192), than those 1–4 years ($2251 to $2521).

**Conclusions:**

Our findings showed a substantial annual RSV-attributable healthcare resource use and costs in the US across age groups, with the highest burden in those aged ≥65 years. These data can be used in cost-effectiveness analyses, and may be useful for policymakers to guide future RSV vaccination and other prevention programs.

## Background

Respiratory syncytial virus (RSV) causes significant morbidity, mortality and healthcare use worldwide, particularly in pre-term infants, children, the elderly and those with pre-existing conditions such as lung and heart disease. [1–5] Globally, it was estimated in 2015, in children < 5 years of age there were 33.1 million episodes of lower respiratory tract infection due to RSV, with 3.2 million RSV-related hospitalizations and 59,600 in-hospital deaths. [6] There are currently no global estimates for RSV infections in other age groups, or in those with underlying medical conditions.

The burden of RSV on the US healthcare system is not clearly established. Based on the limited available data, RSV causes 57,527–132,000 hospitalizations, 1.5–1.7 million office visits and 402,000–517,747 emergency department (ED) visits in children < 5 years of age, [1, 7, 8] and approximately 177,525 hospitalizations each year in those aged ≥65 years. [2] The estimated annual average number of deaths from RSV in the US between 1997 and 2009 was 11,300, [9] with a substantial number in those aged < 5 years and those ≥65 years. [9, 10]

RSV may also increase use of healthcare utilizations through longer-term effects including recurrent wheezing and new diagnosis of asthma, exacerbation of underlying illnesses such as cardiopulmonary conditions and deaths. [2, 11–15]

Given the high burden of RSV, particularly in the young and elderly, and the associated healthcare utilization with infection, it is expected for RSV to lead to substantial healthcare costs. The currently available evidence for RSV-attributable costs is limited by a lack of a control group in many of the studies, and so the impact of RSV on healthcare burden is not clearly defined. In addition, many of these studies in the US focus on infants alone [7, 16–24] and little is known of the impact of RSV for those who are older, such as young children aged between 1 and 4 years and the elderly. [2, 25]

Recent advances in the development of RSV vaccines and monoclonal antibody indicates that the prevention of RSV will likely to be available in the next few years. [26] Understanding healthcare utilization and costs associated with RSV is therefore essential for guiding the implementation of new vaccines and monoclonal antibodies against RSV in various age groups and high-risk populations. [27] The aim of this study was to provide the most recent and comprehensive data on the economic burden of RSV across all ages in the US.

## Methods

### Study design and data source

This retrospective, case-control study used patient data from the Truven Health Marketscan® Commercial Claims and Encounters, and Medicare Supplemental and Coordination of Benefits databases (Truven Health Analytics, Michigan, USA). The Truven Health Marketscan® databases include longitudinal records of patient demographics, inpatient and outpatient services, long-term care accessed, and prescription drug claims covered under a variety of health benefit plans; it is considered representative of the US health population with regards to health coverage [28]. The payments reported in the MarketScan® database represent the amount eligible for payment to providers after applying pricing guidelines such as fee schedules and discounts, and before applying deductibles, copayments, and coordination of benefits.

Medical claims are linked to outpatient prescription drug claims and patient-level enrollment data through the use of unique enrollee identifiers. All database records are de-identified and fully compliant with US patient confidentiality requirements, including the Health Insurance Portability and Accountability Act (HIPAA) of 1996. Because this study used only de-identified patient records, Institutional Review Board (IRB) approval was not required.

### Patient selection

Patients aged ≥1 year with an RSV diagnosis between August 31, 2012 and August 1, 2013 were identified; this time period was chosen in order to cover the RSV epidemic season across the US [29]. We excluded RSV patients aged < 1 year from analysis due to the potential interpretation biases related to missing date of birth and incomplete 1 year of continuous enrollment post-index date in nearly 60% of infants aged < 1 year. An RSV episode was defined by a RSV International Classification of Diseases, 9th revision, Clinical Modification (ICD-9-CM) code (079.6, 466.11 or 480.1) as the principal diagnosis for inpatient admissions, or as the first or secondary diagnosis for outpatient visits, and was considered as first consultation > 28 days following any previous consultation with the same diagnosis code. The index event was defined as the first RSV episode during the 2012–2013 RSV season. Patients were required to have at least 12 months of continuous enrollment pre-index (baseline period) and at least 12 months of continuous enrollment post-index (follow-up period), between August 31, 2011 and August 1, 2014. Patients with a prescription of pavlivizumab, a monoclonal antibody used for the prevention of RSV in high-risk infants, during the study period (baseline or follow-up) were excluded to avoid bias.

Patients with RSV meeting the inclusion criteria were matched 1:1 to controls without RSV based on age, sex, region, health plan, and index date. Controls without RSV were defined as those who did not have a claim associated with ICD-9 codes 079.6, 466.11 or 480.1 in any diagnosis field between August 31, 2012 and August 31, 2014. Controls were also required to have ≥12 months of continuous enrollment both pre- and post-index.

### Baseline measures

Demographic data were collected for the patient on the index date and included; age, age group (1 year, 2–4 years, 5–17 years, 18–49 years, 50–64 years, 65–74 years, 75–84 years, or ≥ 85 years), sex, geographic region (Northeast, North-Central, South, West or unknown), and insurance plan type (commercial or Medicare). Additional descriptive data that were extracted included information on birth prematurity amongst those aged < 5 years, the characteristics of the index RSV episode (setting of the event and whether the event occurred during the epidemic season for the region [29]), history of RSV in the baseline period, and whether the patient had a high-risk medical condition. High-risk medical conditions were categorized based on ICD-9-CM codes for the following conditions: chronic cardiac, pulmonary, renal, metabolic, liver, neurological diseases, diabetes mellitus, hemoglobinopathies, immunosuppressive conditions and malignancy (Appendix 1) [14]. Information on the use of antibiotics and/or antiviral drugs during the baseline and follow-up periods was also extracted. Antibiotics included the following categories: cephalosporins, β-lactams, macrolides, penicillins, tetracyclines, lincosamides, quinolones, trimethoprim-sulfamethoxazole, and any other antibiotic. Antiviral drugs included acyclovir and penciclovir.

### Outcomes measures

All-cause healthcare resource use was assessed in the RSV patients and matched controls over the 12-month follow-up period (from the index event for RSV patients or the index date for respective matched controls). Healthcare resource use included: the percentage of patients with ≥1 claim by service category (inpatient visit, ED and urgent care [UC] visit, ambulatory visit, outpatient visit, or pharmacy prescription), number of visits per category, total length of stay for inpatient visits, and number of prescriptions. Associated costs (including the index event for RSV patients) were computed for each resource category over the 12-month follow-up period. The total unadjusted costs were estimated as the sum of the costs of the individual resource categories for RSV cases and matched controls, respectively. These costs were based on the paid amounts of adjudicated claims, including insurer and health plan payments and patient cost-sharing in the form of copayments, deductibles, and coinsurance. All cost estimates were adjusted to 2014 US dollars using the Medical Care Component of the Consumer Price Index.

### Statistical analysis

Descriptive analyses are presented for the RSV cases and the matched controls. For each continuous variable the mean and standard deviation (SD) were calculated and for each categorical variable the frequency and percentage were calculated. Stratified analyses were conducted by age group (1 year, 2–4 years, 5–17 years, 18–49 years, 50–64 years, 65–74 years, 75–84 years, and ≥ 85 years). The total length of stay in hospital, the number of visits/prescriptions, and the costs for the different resource categories were calculated as a mean per patient taking into account patients without consumption in the calculation. A matched design was used to identify RSV cases and controls and so statistical analyses took into consideration the dependency in the data introduced by matching. Categorical variables were compared using the McNemar test which is a non-parametric test assessing if there is a statistically significant change in proportion for the paired data for dichotomous variables, and the Wilcoxon signed rank test was used for continuous variables to assess if statistically significant difference exists on the median rank of paired-observations. The incremental differences in all-cause resource use and costs between the RSV cases and matched controls were used as an estimate of the RSV-attributable resource use and costs.

Multivariate linear regression models were used to adjust the incremental difference in the total all-cause healthcare costs between cases and controls. Covariates included in the multivariate models were gender, region, high-risk status (at risk if ≥1 high-risk condition during the baseline or follow-up period), history of RSV during the baseline period, and treatments (antibiotics and antivirals). For children aged < 5 years, prematurity were added as additional covariate. Final multivariate models were built using the stepwise selection method (entry level, 0.20; stay level, 0.05).

All analyses were performed using SAS® Enterprise Guide 7.1 (SAS Institute, Cary, NC).

## Results

### Patient characteristics

**Fig. 1:**
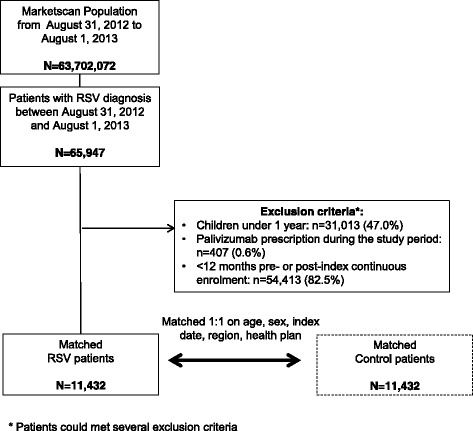
Flow-chart of patient inclusion

Of 63,702,072 individuals enrolled in the databases between August 31, 2012 and August 1, 2013, a total of 11,432 patients with RSV met the inclusion criteria and were matched 1:1 with controls without RSV (Fig. 1).

The two matched populations (RSV cases and controls) had a mean age of 15.8 years (63.2% aged < 5 years), 51.3% were male, and the vast majority were identified from the commercial claims and encounters database (92.8%) (Table 1). In the majority of cases, the index RSV episode occurred during the RSV season (96.5%), with diagnosis in the outpatient setting (80.9%). Significantly more RSV patients had a history of RSV during the baseline period compared to controls (9.2% vs. 1.3%; *p* < 0.0001), and there was a significantly higher proportion of premature patients compared to controls among children aged < 5 years (12.7% vs. 7.5%; *p* < 0.0001) (Table 1).

**Table 1:** Baseline characteristics

Variable	RSV	Control	*p*-value
	(*n* = 11,432)	(*n* = 11,432)	
Mean age, (SD [median])	15.8 (24.5 [2])	15.8 (24.5 [2])	NC
1 year, *n* (%)	4078 (35.7)	4078 (35.7)	
2–4 years, *n* (%)	3142 (27.5)	3142 (27.5)	
5–17 years, *n* (%)	1290 (11.3)	1290 (11.3)	
18–49 years, *n* (%)	1226 (10.7)	1226 (10.7)	
50–64 years, *n* (%)	894 (7.8)	894 (7.8)	
65–74 years, *n* (%)	294 (2.6)	294 (2.6)	
75–84 years, *n* (%)	290 (2.5)	290 (2.5)	
≥ 85 years, *n* (%)	218 (1.9)	218 (1.9)	
Male sex, *n* (%)	5870 (51.3)	5870 (51.3)	NC
Insurance, *n* (%)			
Commercial	10,609 (92.8)	10,609 (92.8)	NC
Medicare	823 (7.2)	823 (7.2)	
US geographic region^a^, *n* (%)			
Northeast	1576 (13.8)	1595 (14.0)	NC
North-central	2804 (24.5)	2803 (24.5)	
South	4775 (41.8)	4734 (41.4)	
West	2114 (18.5)	2137 (18.7)	
Unknown	163 (1.4)	163 (1.4)	
Prematurity,^b^ *n* (%)	914 (12.7)	542 (7.5)	< 0.0001
If preterm, unspecified gestational week, *n* (%)	351 (38.4)	227 (41.9)	
History of RSV during the baseline period, *n* (%)	1055 (9.2)	150 (1.3)	< 0.0001
Index event during the epidemic season, *n* (%)	11,030 (96.5)	–	
Service location of the index event, *n* (%)			
Hospitalization	577 (5.0)	–	
ED/UC	1283 (11.2)	–	
Ambulatory	322 (2.8)	–	
Outpatient	9250 (80.9)	–	

*ED:* emergency department, *NC:* not calculated, *RSV:* respiratory syncytial virus, *UC:* urgent care

^a^ Region: Northeast: Connecticut, Maine, Massachusetts, New Hampshire, Rhode Island, Vermont, New Jersey, New York, Pennsylvania; North-Central: Illinois, Indiana, Michigan, Ohio, Wisconsin, Iowa, Kansas, Minnesota, Missouri, Nebraska, North and South Dakota; South: Delaware, District of Columbia, Florida, Georgia, Maryland, North and South Carolina, Virginia, West Virginia, Alabama, Kentucky, Mississippi, Tennessee, Arkansas, Louisiana, Oklahoma, Texas; West: Arizona, Colorado, Idaho, Montana, Nevada, New Mexico, Utah, Wyoming, Alaska, California, Hawaii, Oregon, Washington

^b^ Percentage among children < 5 years old (*n* = 7220). Prematurity information was not captured in the database for older patients

### High-risk conditions at baseline

**Fig. 2:**
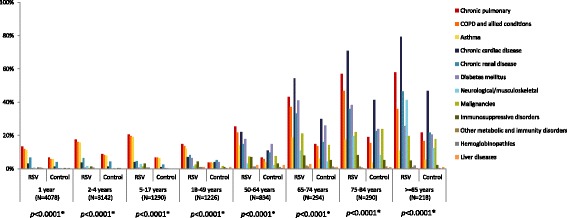
High-risk conditions at baseline in the RSV patients and matched controls. * *P*-values resulting from McNemar’s tests of the proportion of patients with at least one high-risk condition. High-risk conditions were categorized based on ICD-9-CM codes (Appendix 1)

The prevalence of high-risk conditions varied by age for both the RSV and the non-RSV matched control patients (Fig. 2). High-risk conditions were not diagnosed or were infrequent in children and young adults, and were mainly chronic pulmonary diseases. The prevalence of high-risk conditions increased with age, with those aged over 65 years being most commonly affected by comorbidities such as chronic pulmonary, cardiac and renal diseases, and diabetes mellitus (Fig. 2). Malignancies and neurological/musculoskeletal diseases were also most common in those aged ≥75 years. Patients with RSV had more high-risk comorbid conditions than controls. The number of patients with ≥1 high-risk condition was significantly higher in all age groups with RSV compared to matched controls (range 22.3% – 95.9% vs. 10.6% – 73.8%; all *p* < 0.0001; Fig. 2).

### Antibiotic and antiviral treatments

**Fig. 3:**
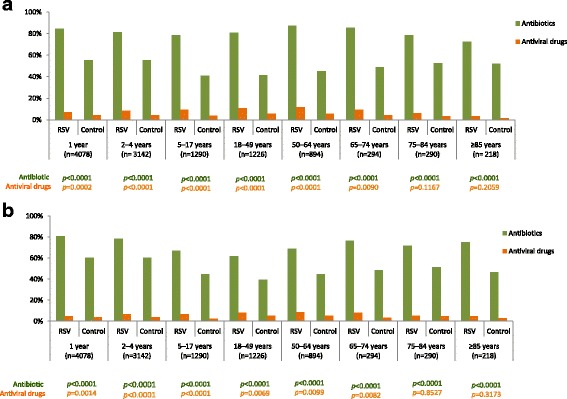
Antibiotics and antiviral drug prescriptions in the baseline period (**a**) and in the follow-up period (**b**)

During the baseline period antibiotic use was higher in the RSV patients (ranging from 61.7% to 80.8%) than matched controls (ranging from 39.6% to 63.8%) (Fig. 3). Antiviral use in the baseline period was low, < 9%, across all ages in both RSV patients and matched controls. During the follow-up period antibiotic use across age groups was similar to that for baseline in matched controls (40.7% to 59.9%), and higher in the RSV patients (72.5% to 87.4%) (Fig. 3). Antiviral use remained low, < 12%, but slightly higher in the RSV patients. During both baseline and follow-up periods, antibiotic use was significantly higher in the RSV patients compared to the matched controls for all age groups (all *p* < 0.0001), and antiviral use was significantly higher in the RSV patients compared to the matched controls for all age groups < 75 years (all *p* < 0.01) (Fig. 3).

### Healthcare resource use and costs

Annual all-cause healthcare resource use in the RSV patients varied by age groups; in the elderly groups (≥65 years) healthcare resource use was higher (43.2%–60.6% with ≥1 hospitalization; 47.6%–65.1% with ≥1 ED/UC visit; 76.1%–82.1% with ≥1 ambulatory visit) than in children and adults (9.5%–18.2% with ≥1 hospitalization; 32.0%–46.9% with ≥1 ED/UC visit; 35.1%–66.1% with ≥1 ambulatory visit) (Table 2). The greatest resource use was seen for outpatient visits with > 98% across all age-groups for the RSV patients having at least one visit. Annual healthcare resource use was significantly higher among the RSV patients compared to the matched control patients across all age-groups and resource categories both with respect to units used (all *p* < 0.0001) and frequency of use (all *p* < 0.05, except for prescription in elderly aged ≥75 years) (Table 2). Using incremental differences between the RSV patients and matched controls, an important part of healthcare resource use can be attributable to RSV; and this contribution varied across each age group. The greatest incremental differences in healthcare resource use between the RSV patients and matched controls were observed in the elderly; for ambulatory visits, outpatient visits, and prescriptions filled, the incremental differences increased with age (Table 3).

**Table 2:** Annual healthcare utilization and costs among patients with RSV and matched controls during the follow-up period

Resource category	1 year			2–4 years			5–17 years		
	RSV (*n* = 4078)	Matched controls (*n* = 4078)	*p*-value	RSV (*n* = 3142)	Matched controls (*n* = 3142)	*p*-value	RSV (*n* = 1290)	Matched controls (*n* = 1290)	*p*-value
Inpatient visit									
≥ 1 visit, *n* (%)	521 (12.8)	89 (2.2)	< 0.0001	392 (12.5)	47 (1.5)	< 0.0001	123 (9.5)	23 (1.8)	< 0.0001
No. of visits, mean (SD)	0.2 (0.6)	0 (0.2)	< 0.0001	0.2 (0.6)	0 (0.4)	< 0.0001	0.2 (0.8)	0 (0.2)	< 0.0001
Length of stay (days), mean (SD)	0.4 (2.1)	0 (0.7)	< 0.0001	0.4 (2.1)	0.1 (1.9)	< 0.0001	0.7 (4.3)	0 (0.5)	< 0.0001
Inpatient costs, mean (SD)	$1625 ($11,249)	$280 ($6376)	< 0.0001	$2016 ($14,192)	$379 ($11,230)	< 0.0001	$4325 ($43,249)	$470 ($7593)	< 0.0001
ED/UC									
≥ 1 visit, *n* (%)	1912 (46.9)	1079 (26.5)	< 0.0001	1368 (43.5)	741 (23.6)	< 0.0001	413 (32.0)	217 (16.8)	< 0.0001
No. of visits, mean (SD)	0.5 (0.6)	0.3 (0.5)	< 0.0001	0.5 (0.6)	0.2 (0.5)	< 0.0001	0.4 (0.7)	0.2 (0.4)	< 0.0001
ED/UC costs, mean (SD)	$789 ($1926)	$298 ($915)	< 0.0001	$712 ($1597)	$273 ($1068)	< 0.0001	$644 ($2040)	$177 ($742)	< 0.0001
Ambulatory visit									
≥ 1 visit, *n* (%)	1784 (43.7)	954 (23.4)	< 0.0001	1280 (40.7)	704 (22.4)	< 0.0001	453 (35.1)	258 (20.0)	< 0.0001
No. of visits, mean (SD)	1.3 (4.5)	0.6 (2.8)	< 0.0001	1.5 (5.8)	0.7 (3.8)	< 0.0001	1.7 (5.4)	0.5 (2.7)	< 0.0001
Ambulatory costs, mean (SD)	$1270 ($5866)	$557 ($3735)	< 0.0001	$1425 ($6179)	$693 ($6427)	< 0.0001	$2107 ($10,423)	$327 ($1733)	< 0.0001
Outpatient visit									
≥ 1 visit, *n* (%)	4065 (99.7)	3920 (96.1)	< 0.0001	3126 (99.5)	2975 (94.7)	< 0.0001	1280 (99.2)	1107 (85.8)	< 0.0001
No. of visits, mean (SD)	11 (10.6)	6 (7.7)	< 0.0001	10.1 (15.5)	5.4 (10.0)	< 0.0001	11 (20.4)	4.8 (9.7)	< 0.0001
Outpatient costs, mean (SD)	$1615 ($3480)	$777 ($1278)	< 0.0001	$1692 ($7748)	$843 ($3224)	< 0.0001	$2537 ($12,573)	$713 ($3115)	< 0.0001
Prescriptions filled									
≥ 1 prescription, *n* (%)	3863 (94.7)	2943 (72.2)	< 0.0001	2966 (94.4)	2227 (70.9)	< 0.0001	1197 (92.8)	802 (62.2)	< 0.0001
No. of prescriptions, mean (SD)	5.8 (5.5)	2.7 (3.5)	< 0.0001	6.0 (6.9)	2.6 (3.7)	< 0.0001	7.2 (10.2)	2.9 (6.0)	< 0.0001
Prescription costs, mean (SD)	$444 ($913)	$158 ($408)	< 0.0001	$708 ($3798)	$193 ($875)	< 0.0001	$1399 ($6765)	$529 ($4253)	< 0.0001
Total unadjusted costs,^a^ mean (SD)	$5742 ($17,282)	$2069 ($9562)	< 0.0001	$6553 ($22,881)	$2380 ($18,909)	< 0.0001	$11,013 ($60,208)	$2216 ($10,908)	< 0.0001
Resource category		18–49 years				50–64 years			
		RSV (*n* = 1226)	Matched controls (*n* = 1226)	*p*-value		RSV (*n* = 894)	Matched controls (*n* = 894)	*p*-value	
Inpatient visit									
≥ 1 visit, *n* (%)		153 (12.5)	77 (6.3)	< 0.0001		163 (18.2)	74 (8.3)	< 0.0001	
No. of visits, mean (SD)		0.2 (0.6)	0.1 (0.3)	< 0.0001		0.3 (0.7)	0.1 (0.5)	< 0.0001	
Length of stay (days), mean (SD)		0.6 (4.2)	0.2 (1.3)	< 0.0001		1.1 (5.3)	0.3 (2.2)	< 0.0001	
Inpatient costs, mean (SD)		$3416 ($25,766)	$1032 ($7111)	< 0.0001		$5309 ($30,503)	$2396 ($29,349)	< 0.0001	
ED/UC									
≥ 1 visit, *n* (%)		461 (37.6)	241 (19.7)	< 0.0001		325 (36.4)	154 (17.2)	< 0.0001	
No. of visits, mean (SD)		0.4 (0.6)	0.2 (0.4)	< 0.0001		0.4 (0.7)	0.2 (0.5)	< 0.0001	
ED/UC costs, mean (SD)		$881 ($3634)	$280 ($1041)	< 0.0001		$1160 ($4940)	$334 ($1420)	< 0.0001	
Ambulatory visits									
≥ 1 visit, *n* (%)		600 (48.9)	450 (36.7)	< 0.0001		591 (66.1)	528 (59.1)	0.0017	
No. of visits, mean (SD)		2.1 (6.0)	1.1 (2.5)	< 0.0001		3.6 (7.1)	2.2 (4.8)	< 0.0001	
Ambulatory costs, mean (SD)		$2607 ($12,359)	$1082 ($3910)	< 0.0001		$4524 ($17,347)	$2350 ($8518)	< 0.0001	
Outpatient visits									
≥ 1 visit, *n* (%)		1212 (98.9)	1012 (82.5)	< 0.0001		893 (99.9)	818 (91.5)	< 0.0001	
No. of visits, mean (SD)		12.3 (15.0)	6.8 (10.4)	< 0.0001		17.8 (18.1)	9.9 (11.4)	< 0.0001	
Outpatient costs, mean (SD)		$2417 ($7599)	$1223 ($5698)	< 0.0001		$3455 ($6481)	$1860 ($6240)	< 0.0001	
Prescriptions filled									
≥ 1 prescription, *n* (%)		1148 (93.6)	930 (75.9)	< 0.0001		859 (96.1)	766 (85.7)	< 0.0001	
No. of prescriptions, mean (SD)		11.3 (13.1)	7 (8.8)	< 0.0001		20.4 (17.5)	12.9 (13.9)	< 0.0001	
Prescription costs, mean (SD)		$1861 ($6837)	$733 ($2239)	< 0.0001		$3477 ($6789)	$2231 ($6284)	< 0.0001	
Total unadjusted costs^a^, mean (SD)		$11,182 ($42,469)	$4350 ($11,749)	< 0.0001		$17,925 (46,222)	$9172 ($38,563)	< 0.0001	
Resource category	65–74 years			75–84 years			≥85 years		
	RSV (*n* = 294)	Matched controls (*n* = 294)	*p*-value	RSV (*n* = 290)	Matched controls (*n* = 290)	*p*-value	RSV (*n* = 218)	Matched controls (*n* = 218)	*p*-value
Inpatient visit									
≥ 1 visit, *n* (%)	127 (43.2)	60 (20.4)	< 0.0001	157 (54.1)	90 (31.0)	< 0.0001	132 (60.6)	70 (32.1)	< 0.0001
No. of visits, mean (SD)	0.8 (1.1)	0.3 (0.6)	< 0.0001	1.1 (1.3)	0.5 (0.8)	< 0.0001	1.0 (1.0)	0.5 (1.0)	< 0.0001
Length of stay (days), mean (SD)	3.4 (10.0)	0.5 (1.8)	< 0.0001	4.2 (7.8)	1.2 (3.3)	< 0.0001	3.6 (6.1)	1.7 (4.5)	< 0.0001
Inpatient costs, mean (SD)	$8433 ($22,394)	$1981 ($6785)	< 0.0001	$16,286 ($69,695)	$4903 ($17,511)	< 0.0001	$7113 ($12,020)	$4119 ($16,732)	< 0.0001
ED/UC									
≥ 1 visit, *n* (%)	140 (47.6)	73 (24.8)	< 0.0001	171 (59.0)	107 (36.9)	< 0.0001	142 (65.1)	84 (38.5)	< 0.0001
No. of visits, mean (SD)	0.7 (0.9)	0.3 (0.5)	< 0.0001	1.0 (1.2)	0.5 (0.8)	< 0.0001	1.0 (1.0)	0.6 (0.9)	< 0.0001
ED/UC costs, mean (SD)	$916 ($4745)	$257 ($885)	< 0.0001	$1412 ($3404)	$692 ($2126)	< 0.0001	$1370 ($3687)	$657 ($1790)	0.0006
Ambulatory visits									
≥ 1 visit, *n* (%)	231 (78.6)	194 (66.0)	0.0007	238 (82.1)	201 (69.3)	0.0004	166 (76.1)	139 (63.8)	0.0046
No. of visits, mean (SD)	6 (9.8)	3.8 (6.5)	< 0.0001	6.6 (10.2)	3.9 (7.4)	< 0.0001	5.2 (7.7)	4.5 (9.6)	< 0.0001
Ambulatory costs, mean (SD)	$4257 ($15,648)	$2660 ($6004)	0.0296	$5033 ($16,104)	$4019 ($22,583)	0.0033	$5764 ($44,448)	$1915 ($5273)	0.0093
Outpatient visits									
≥ 1 visit, *n* (%)	293 (99.7)	277 (94.2)	0.0002	289 (99.7)	272 (93.8)	< 0.0001	217 (99.5)	202 (92.7)	0.0003
No. of visits, mean (SD)	25.1 (22.0)	13.0 (15.5)	< 0.0001	35.3 (32.8)	17.2 (19.3)	< 0.0001	35.7 (29.7)	17.1 (17.2)	< 0.0001
Outpatient costs, mean (SD)	$6957 ($12,526)	$2637 ($6586)	< 0.0001	$16,627 ($52,729)	$4842 ($12,823)	< 0.0001	$13,002 ($17,381)	$4604 ($9504)	< 0.0001
Prescriptions filled									
≥ 1 prescription, *n* (%)	282 (95.9)	268 (91.2)	0.0164	274 (94.5)	272 (93.8)	0.7237	200 (91.7)	199 (91.3)	0.8575
No. of prescriptions, mean (SD)	27.0 (21.1)	17.0 (14.1)	< 0.0001	31.2 (23.9)	21.7 (15.8)	< 0.0001	38.2 (29.1)	23.6 (19.9)	< 0.0001
Prescription costs, mean (SD)	$4866 ($7563)	$2844 ($6288)	< 0.0001	$4204 ($4644)	$2955 ($6331)	< 0.0001	$2958 ($2972)	$2159 ($3690)	< 0.0001
Total unadjusted costs^a^, mean (SD)	$25,429 ($38,653)	$10,379 ($16,103)	< 0.0001	$43,562 ($99,939)	$17,412 ($36,723)	< 0.0001	$30,206 ($56,549)	$13,454 ($23,848)	< 0.0001

*ED:* emergency department, *RSV:* respiratory syncytial virus, *UC:* urgent care

Costs adjust to 2014 US dollars

^a^Total unadjusted costs are the sum of the costs from the individual resource categories

**Table 3:** Incremental differences in annual healthcare utilization and costs between the RSV patients and the matched controls over the follow-up period

	1 year	2–4 years	5–17 years	18–49 years	50–64 years	65–74 years	75–84 years	≥85 years
Inpatient visit								
≥ 1 visit, (%)	10.6	11	7.7	6.2	9.9	22.8	23.1	28.5
No. of visits, mean	0.2	0.2	0.2	0.1	0.2	0.5	0.6	0.5
Length of stay (days), mean	0.4	0.3	0.7	0.4	0.8	2.9	3.0	1.9
Inpatient costs, mean	$1345	$1637	$3855	$2384	$2913	$6453	$11,383	$2993
ED/UC								
≥ 1 visit, (%)	20.4	19.9	15.2	17.9	19.2	22.8	22.1	26.6
No. of visits, mean	0.2	0.3	0.2	0.2	0.2	0.4	0.5	0.4
ED/UC costs, mean	$491	$440	$467	$601	$826	$658	$720	$713
Ambulatory visit								
≥ 1 visit, (%)	20.3	18.3	15.1	12.2	7.0	12.6	12.8	12.3
No. of visits, mean	0.7	0.8	1.2	1.0	1.4	2.2	2.7	0.7
Ambulatory costs, mean	$713	$732	$1780	$1525	$2174	$1597	$1014	$3849
Outpatient visit								
≥ 1 visit, (%)	3.6	4.8	13.4	16.4	8.4	5.5	5.9	6.8
No. of visits, mean	5.0	4.7	6.2	5.5	7.9	12.1	18.1	18.6
Outpatient costs, mean	$839	$849	$1824	$1194	$1595	$4320	$11,785	$8398
Prescriptions filled								
≥ 1 prescription, (%)	22.5	23.5	30.6	17.7	10.4	4.7	0.7	0.4
No. of prescriptions, mean	3.1	3.4	4.3	4.3	7.5	10.0	9.5	14.6
Prescription costs, mean	$285	$515	$870	$1127	$1246	$2022	$1249	$800
Total unadjusted costs,^a^ mean	$3673	$4173	$8796	$6832	$8753	$15,050	$26,151	$16,752

*ED:* emergency department, *RSV:* respiratory syncytial virus, *UC:* urgent care

Costs adjusted to 2014 US dollars

^a^Total unadjusted costs are the sum of the costs from the individual resource categories

**Fig. 4:**
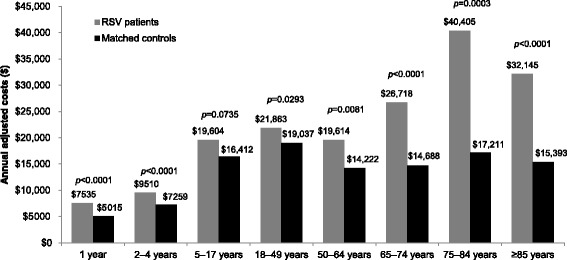
Adjusted annual healthcare costs among patients with RSV and matched controls during the follow-up period. Costs adjusted to 2014 US dollars. Multivariate linear regression models were used to adjust the total healthcare costs; covariates included gender, region, high-risk status, history of RSV during the baseline period, treatments and prematurity for children aged < 5 years

Multivariate analyses incorporating confounding factors showed significantly higher adjusted total annual costs in the RSV patients compared to the matched controls across all age groups; with the adjusted total annual costs in the RSV patients ranging from $7535 (1 year group) to $40,405 (75–84 years group) and in the matched controls from $5015 (1 year group) to $19,037 (75–84 years group) (Fig. 4). The incremental difference between RSV and matched controls, as an estimate of the annual adjusted costs attributable to RSV, were higher in the elderly than in adults and children; $2521, $2251, $3192, $2825, $5391, $12,030, $23,194, $16,752 for the 1 year, 2–4 years, 5–17 years, 18–49 years, 50–64 years, 65–74 years, 75–84 years and ≥ 85 years age groups, respectively.

## Discussion

This is the first comprehensive study of annual healthcare resource use and costs in those with RSV across multiple age groups in the US. Through the use of matched control patients without RSV we were also able to estimate the incremental differences in resource use and costs between those with and without RSV. There was higher annual resource use in the RSV patients across all age groups; when compared to non-RSV matched controls, up to 29% more in the RSV group had ≥1 hospitalization, up to 27% more in the RSV group had ≥1 ED/UC visits and up to 20% more in the RSV group had ≥1 ambulatory visit. The adjusted annual costs for those with an RSV event were also higher when compared to matched controls across all age groups, with incremental differences of $2251 to $23,194. Due to the matching of RSV and non-RSV patients, which controlled for some confounding factors, we can conclude that this higher resource use and higher costs are related to RSV.

Previous studies of healthcare costs related to RSV in the US have generally focused on infants, often comparing full-term and pre-term infants. These studies highlight the higher burden of RSV in preterm infants, with annual hospitalization costs as high as $19,559 to $52,900. [16, 19, 22] In studies in infants with matched controls, those with RSV had higher rates of hospitalization, higher ED use, more ambulatory visits, more prescriptions filled and more antibiotic use. [17, 18, 22] In a study of costs in children aged < 5 years in the US, annual mean hospitalization costs in those with RSV was $4584, with total estimated medical costs of RSV of $341–449 million, with a further $258 million estimated for ambulatory care costs. [7] In our study only those ≥1 year old were included in this analysis. We were also interested in healthcare utilization and costs in infants aged < 1 year, due to the documented burden of RSV in this population, and particularly wished to explore outcomes by age subgroups (< 3 months, 3–5 months, and 6–11 months). However, among the 31,013 infants aged < 1 year with a RSV diagnosis during the 2012–2013 season, the results were not coherent and did not follow the global trend of healthcare costs. We were not able to identify the potential bias or confounding factors for these unexpected results, and so they were not included in the final analyses. One source of the potential bias may have been related to the process used to retrieve the birth date, as date of birth is not available in the MarketScan® databases; the date of the first claim with an ICD-9-CM code for birth was used as a proxy for birth date (Appendix 2), if this was not available then the first enrolment date was used when year of enrolment and birth year were the same. In the process of identifying patients for inclusion, 16.3% (*n* = 5054) did not have an estimable date of birth and 52.3% (*n* = 16,390) did not have 1 year of continuous enrollment post-index date in infants aged < 1 year. These issues might have contributed to these unexpected results, or by the step of matching controls in this group.

In studies of RSV-attributable healthcare costs in the elderly in the US, estimates have been made of annual hospitalization costs of $11,000 per hospitalization, with total costs for all elderly aged ≥65 years estimated from $150–680 million [25] to $1 billion. [2] In a study of all adults in the US, mean adjusted costs for RSV hospitalization across age groups was calculated as $38,828. [30] However, that study found the highest hospitalization costs in those aged 45–59 years, and lowest in those ≥60 years; [30] this differs to the findings in our study, where the highest resource use and costs were seen in those aged ≥65 years. Compared to those who were aged 18–49 years, in those ≥65 years, 3–5 times more patients had a hospitalization, 1–2 times more had an ED/UC visit, and twice as many had an ambulatory visit, with a higher mean number of visits for each setting and longer hospital stays. This higher resource use also translated to higher costs, with the highest adjusted annual healthcare costs seen in those with RSV who were aged 75–84 years, almost double the costs seen in those aged 18–49 years. The highest incremental difference in costs between the RSV and matched-control group was also seen in the elderly patients.

The higher resource use and costs in the RSV patients, beyond the costs directly related to the RSV event itself, may be due to the complications and long-term effects that have been suggested to result from RSV infection, such as wheezing, asthma and allergies in children [11, 17, 18, 20], and exacerbation of underlying conditions, such as pulmonary or cardiovascular diseases. [14, 20]

This study also showed a substantial increase in the number of antibiotic prescriptions in patients in the RSV group in the year after RSV infection compared to the baseline period and to those in the non-RSV matched control group. This increase in antibiotic prescriptions may have been due to the inappropriate use of antibiotics for RSV, or may be a further indication of the longer-term impact of RSV. Previous studies have also shown a high level of unnecessary antibiotic use in RSV, [12, 13, 31, 32] with estimates of excess antibiotic prescriptions in RSV of 641 per 1000 in children aged < 5 years in the US. [13] Bacteremia is rarely concurrent with RSV infections, even in those with high-risk for infection, [32, 33] and as such antibiotic use has no clinical benefit and leads to increased costs and the potential for increase in drug resistance. [31]

Generating comprehensive data on healthcare resource use and costs associated with RSV across several age groups will help to provide valuable information for the development of cost-effectiveness models, and help guide prevention strategies against RSV. Protective immunity to RSV induced by natural infection is weak and short-lived, [34, 35] and current prevention strategies are restricted to at-risk infants with repeated doses of the expensive monoclonal antibody palivizumab during the RSV season. However, the cost-effectiveness of palivizumab has not been definitively proven, with studies both for and against its cost-effectiveness. [36] Consequently, new approaches for preventing and treating RSV that are also cost-effective, particularly for at-risk groups such as infants, young children, elderly and those with underlying medical conditions, are needed. Vaccines against RSV are currently in development, with phase 2 and 3 studies ongoing for one vaccine, including for the vaccination of pregnant women in order to confer immunity to newborns. [37, 38] In addition, a late stage phase 2b trial of a highly potent monoclonal antibody, MEDI8897, in pre-term infants is expected to complete in 2018, [39] with plans for a phase 3 study in healthy full-term and late pre-term infants. Current data on RSV infections rates has allowed for modeling studies to identify children aged < 5 years as those most likely to be infected with RSV, and most likely to transmit it, [40] supporting previous suggestion that children are an important source of RSV transmission to adults, [41] consequently those aged < 5 years are the target group for future vaccination. Our data on the economic impact of RSV across age groups can be used to re-evaluate cost effectiveness of new vaccines or monoclonal antibodies, especially in the elderly age groups.

This was a large observational study of over 11,000 patients ≥1 year of age with an RSV event in the US, and with follow-up data for 12 months. This analysis, covering a wide range of ages, allowed assessment of RSV-related costs in children, adults and the elderly; utilizing age ranges chosen to focus on those age groups with the highest risk. The use of matched non-RSV controls allowed for comparison and estimates of the RSV-attributable resource use and costs, which is lacking in many other RSV costs studies. There were, however, several limitations to this analysis. ICD-9-CM codes were used to identify RSV cases and healthcare resource use and costs in the 12 month follow-up period; miscoding or misclassification or missed opportunities for RSV testing may have occurred leading to a misdiagnosis or incorrect utilization or cost to be attributed. Similarly, where a patient did not make an insurance claim the associated healthcare resource use or cost would not have been captured in the database. Some outcomes that may be relevant to this analysis, such as mortality or the severity of disease were not available. The completeness of the data on prematurity in those aged < 5 years in the databases is uncertain; prematurity was identified using ICD-9CM and additional diagnosis-related group codes (Appendix 3), however, the proportion of premature child who are not identified by these codes is unknown. The severity of prematurity was also not assessable for almost 40% of premature children in this study. Palivizumab use during hospitalization was not captured in the databases, so not all patients with palivizumab would have been excluded from the analysis. Only direct medical costs were captured in this analysis, other economic consequences such as out-of-pocket costs or loss of productivity were not included. Assessment of out-of-pocket costs in infants with RSV have been estimated to be between $214–644, with $1921–3873 estimated in lost productivity. [24] The data presented here may not be applicable to the entire US population, as this analysis is based on a commercially insured population.

## Conclusions

This study presents the annual healthcare resource use and costs of RSV in the US across age groups, highlighting the particular burden in those aged ≥65 years. These data confirm the significant healthcare burden of RSV and need for the prevention of RSV to be a high priority across age groups, and that additional safe and effective preventative measures are required.

## Appendix 1

**Table 4:** High-risk conditions ICD-9-CM codes

HIGH RISK CATEGORY	HIGH-RISK SUB-CATEGORY	ICD-9-CM CODES
Chronic cardiac disease	Acute rheumatic fever	391–392
	Chronic rheumatic heart disease	393–398
	Hypertensive heart disease	402, 404
	Ischemic heart disease	410–414
	Diseases of pulmonary circulation	416, 417
	Other forms of heart disease	421, 423, 424, 425, 427.1–427.5, 427.8, 428, 429
	Atherosclerosis, polyarteritis nodosa	440, 446
	Congenital anomalies	745–747
	Surgical/device conditions	V42.1, V45.0, V45.81, V45.82
	Cardiovascular syphilis	093
	Candidal endocarditis	11,281
	Myocarditis due to toxoplasmosis	1303
Chronic pulmonary	Other metabolic and immunity disorders	277.0, 277.6
	COPD and allied conditions	491–496
	Pneumoconioses/other lung diseases due to external agents	500–506, 507.0, 507.1, 508
	Other diseases of respiratory system	510, 513–517, 518.0–518.3, 519.0, 519.9
	Congenital anomalies	748.4–748.6, 759.3
	Lung transplant	V426
	Tuberculosis	011, 012
	Diseases due to other mycobacteria	031.0
	Sarcoidosis	135
Chronic renal disease	Hypertensive renal disease	403
	Nephritis, nephrotic syndrome, nephrosis	581–583, 585–587, 588.0, 588.1
	Chronic pyelonephritis	590.0
	Other specified disorders of kidney and ureter	593.8
	Dialysis and transplant	V42.0, V45.1, V56
Diabetes mellitus	Diabetes mellitus	250, 251, 648.0
	Complications of diabetes	357.2, 362.0, 362.11, 366.41
Hemoglobinopathies	Anemias	282–284
Immunosuppressive disorders	HIV/retroviral disease	042–044, 079.5, V08
	Disorders involving immune mechanism	279
	Diseases of blood and blood-forming organs	288.0, 288.1, 288.2
	Polyarteritis nodosa	446
	Diseases of musculoskeletal system and connective tissue	710.0, 710.2, 710.4, 714
	Organ/tissue transplants	V420–V422, V426–V429
	Radiation/chemotherapy	V580, V581
Malignancies		140–208
Other metabolic and immunity disorders	Disorders of adrenal glands	255
	Other disorders	270, 271, 277.2, 277.3, 277.5, 277.8
Liver diseases	Chronic liver disease and cirrhosis	571
	Liver abscess and sequelae of chronic liver disease	572.1–572.8
Neurological/ musculoskeletal	Psychotic conditions	290, 294.1
	Mental retardation	318.1, 318.2
	Hereditary and degenerative diseases of CNS	330, 331, 333.0, 333.4–333.9, 334, 335
	Other disorders of CNS	340, 341, 343, 344.0
	Disorders of peripheral nervous system	358.0, 358.1, 359.1, 359.2
	Late effects of CVD	438
	Chondrodystrophy	756.4

## Appendix 2

**Table 5:** ICD9 codes for confirmation of birth

CODE TYPE	CODE	MEDICAL CODING
ICD-9-CM Diagnosis	V27	Outcome of delivery
	V30	Single liveborn
	V31	Twin birth mate liveborn
	V32	Twin birth mate stillborn
	V33	Twin birth unspecified whether mate liveborn or stillborn
	V34	Other multiple birth (three or more) mates all liveborn
	V35	Other multiple birth (three or more) mates all stillborn
	V36	Other multiple birth (three or more) mates liveborn and stillborn
	V37	Other multiple birth (three or more) unspecified whether mates liveborn or stillborn
	V39	Liveborn unspecified whether single twin or multiple
	650–659	Normal Delivery, And Other Indications For Care In Pregnancy, Labor, And Delivery
	660–669	Complications Occurring Mainly In The Course Of Labor And Delivery
	763	Fetus or newborn affected by other complications of labor and delivery
	765	Disorders relating to short gestation and unspecified low birthweight
	766	Disorders relating to long gestation and high birthweight
	767	Birth trauma
CPT codes	99,460	Initial hospital or birthing center care, per day, for evaluation and management of normal newborn infant
	99,461	Initial care, per day, for evaluation and management of normal newborn infant seen in other than hospital or birthing center
	99,462	Subsequent hospital care, per day, for evaluation and management of normal newborn
	99,463	Initial hospital or birthing center care, per day, for evaluation and management of normal newborn infant admitted and discharged on the same date
DRG codes	789	NEONATES DIED OR TRANS TO AC CARE FAC
	790	EXTR IMMATURITY/RESP DIST SYND, NEONATE
	791	PREMATURITY W MAJOR PROBLEMS
	792	PREMATURITY WO MAJOR PROBLEMS
	793	FULL TERM NEONATE W MAJOR PROBLEMS
	794	NEONATE WI OTH SIGNIFICANT PROBLEMS
	795	NORMAL NEWBORN

## Appendix 3

**Table 6:** ICD9 codes for prematurity

CODE TYPE	CODE	MEDICAL CODING
ICD-9-CM Diagnosis	765.0X	Disorders relating to extreme immaturity of infant
	765.1	Disorders relating to other preterm infants
	765.2	Weeks of gestation

## Abbreviations

ED: Emergency department
ICD-9-CM: International Classification of Diseases, 9th revision, Clinical Modification
RSV: Respiratory syncytial virus
UC: Urgent care
